# Mental Health Problems among Adolescents with Early-Onset and Long-Duration Type 1 Diabetes and Their Association with Quality of Life: A Population-Based Survey

**DOI:** 10.1371/journal.pone.0092473

**Published:** 2014-03-17

**Authors:** Anna Stahl-Pehe, Karin Lange, Christina Bächle, Katty Castillo, Reinhard W. Holl, Joachim Rosenbauer

**Affiliations:** 1 Institute for Biometrics and Epidemiology, German Diabetes Center (DDZ), Leibniz Center for Diabetes Research at the Heinrich Heine University, Düsseldorf, Germany; 2 Medical Psychology Unit, Hannover Medical School, Hannover, Germany; 3 Institute of Epidemiology and Medical Biometry, University of Ulm, Ulm, Germany; Endocrine Research Center (Firouzgar), Institute of Endocrinology and Metabolism, Islamic Republic of Iran

## Abstract

**Objective:**

To evaluate mental health problems and associations between mental health problems and health-related quality of life in adolescents with type 1 diabetes in comparison with the general population.

**Method:**

A total of 629 11- to 17-year-olds with early-onset and long-lasting type 1 diabetes and their parents completed comprehensive questionnaires. Mental health was assessed using the parent- and self-report versions of the Strengths and Difficulties Questionnaire (SDQ). The Revised Children's Quality of Life Questionnaire (KINDL-R) was used to measure quality of life. The comparison group (n = 6,813) was a representative sample from the German KiGGS study.

**Results:**

The proportion of youths with mental health problems (defined as abnormal SDQ total difficulties score) was, based on self-reports, 4.4% in the patient group and 2.9% in the general population (adjusted OR = 1.61, p = 0.044); and based on proxy reports, 7.9% in the patient group and 7.2% in the general population (OR = 1.05, p = 0.788). Youths with type 1 diabetes and self-reported mental health problems scored worse in the KINDL-R subscales of physical well-being (adjusted average difference β = −16.74, p<0.001) and family (β = −11.09, p = 0.017), and in the KINDL-R total score (β = −8.09, p<0.001), than peers with self-reported mental health problems. The quality of life of diabetic adolescents and proxy-reported mental health problems did not differ from peers with proxy-reported mental health problems adjusted for confounders.

**Conclusions:**

Compared with the general population with mental health problems, the quality of life of adolescents with type 1 diabetes who report mental health problems is more severely impaired. This observation calls for early prevention and intervention as part of pediatric diabetes long-term care.

## Introduction

Type 1 diabetes is a chronic disease that results from autoimmune destruction of insulin-producing beta cells of the pancreas. Optimal insulin substitution is necessary to achieve near-normal glycemic control and to avoid serious short- and long-term complications. In recent years, biomedical and technological advances have been made in the treatment of type 1 diabetes. Children and adolescents in Western countries have benefited from these developments in that flexible insulin regimens allow to lead a more normal life. However, the therapeutic options raise rather than supersede behavioral and psychological aspects of diabetes care [1]. In Germany, structured patient education programs are regarded as an indispensable part of diabetes treatment. The aim of these patient education programs is to aid patients in successfully managing their therapy to avoid negative acute and long-term complications and to maintain a high quality of life (QoL) [2]. Glycemic control has improved in children and adolescents with type 1 diabetes in Germany during the past decade [3], but everyday practice of complex treatment regimens remains challenging for patients and their parents. Behavioral and psychosocial aspects of diabetes care in adolescents with type 1 diabetes are particularly concerning because mental health issues can interfere with diabetes self-management (e.g., self-monitoring of blood glucose levels and injecting insulin as recommended). Adolescents with type 1 diabetes and mental health comorbidities are at increased risk of having less effective self-care, poor glycemic control, and poorer health outcomes. Thus, mental health problems in adolescence may have a potentially persistent impact on the life of the patients [4], [5]. Several studies have been conducted with regard to mental health problems in adolescents with type 1 diabetes [5]–[10]. However, none of these studies focused specifically on patients with type 1 diabetes onset during the first five years of life, who suffer from the disease for nearly all of their lives. This is surprising because the incidence of early-onset type 1 diabetes has been steeply increasing in several Western countries for some years [11]. In addition, there is increasing evidence that the individual and societal impact in patients with early disease onset is higher than in patients with later onset [12], [13].

As yet, it is not entirely clear whether mental health is worse in adolescents with type 1 diabetes than in the general population. It is assumed that adolescents with type 1 diabetes have an increased risk of cognitive dysfunction (including attention problems), which is likely a consequence of structural and functional changes to the brain (most apparent in those patients with early-onset type 1 diabetes) [14]. It has been observed that the parent-reported mental health of children and adolescents with type 1 diabetes is poorer than the mental health of normative non-clinical samples [5], [6]. However, some studies based on self-reports [6]–[9] and parent-reports [10] have shown that mental health problems (including psychosocial difficulties and behavioral problems) are no more prevalent in adolescents with type 1 diabetes than in healthy control subjects. This circumstance led to our first research question: *Do mental health problems occur more frequently in adolescents with early-onset type 1 diabetes than in the general population?*

There is some evidence that mental health problems are associated with reduced QoL in the general population of children and adolescents [15]–[17], although another report did not describe an association between mental health problems and QoL [18]. QoL is an important outcome of pediatric diabetes care [19]. We observed in a previous analysis that QoL was not impaired in adolescents with early-onset type 1 diabetes compared with peers from the general population in Germany [20]. However, adolescents with type 1 diabetes and mental health problems might be a sub-group with increased risk of impaired QoL. There is a lack of studies on this topic to date. This leads us to our second research question: *Is the QoL of adolescents with early-onset type 1 diabetes and mental health problems worse than the QoL of peers from the general population with mental health problems?*

## Research Design and Methods

### Data sources

One data source was the nationwide, population-based questionnaire survey “Clinical Course of Type 1 Diabetes in Children, Adolescents and Young Adults with Disease Onset in Preschool Age” (diabetes study). The patients were selected from the nationwide diabetes register maintained at the German Diabetes Center, Düsseldorf, Germany, in cases where the type 1 diabetes onset was during the period of 1993 to 1999 when the patients were younger than 5 years old. The study was approved by the responsible commissioner for data protection and the ethics committee of Düsseldorf University (study number 3254). Data were collected via standardized comprehensive self-administered questionnaires during the years 2009 and 2010. The patients and their parents/caregivers (proxies) gave written informed consent and answered the mailed questionnaires at home. The response rate was 43% among 11- to 13-year-olds and 42% among 14- to 17-year-olds. The study is described in more detail elsewhere [20], [21].

The second data source was the Public Use File of the German Health Interview and Examination Survey for Children and Adolescents (KiGGS) conducted from 2003 to 2006 (Robert Koch Institute, Berlin (Germany), 2008). KiGGS is a nationwide survey, representative for non-institutionalized children and adolescents living in Germany. The data collection included questionnaires that were answered by 11- to 17-year-olds and their parents at the study centers. The response rate was 69% in 11- to 13-year-olds and 63% in 14- to 17-year-olds [22]. The extensive, standardized questionnaires used in the diabetes study were to a large extent identical to those used in the KiGGS, but contained additional diabetes-specific questions.

### The study population

A total of 629 diabetic children and adolescents fulfilled the inclusion criteria for the patient group: they had been newly diagnosed with type 1 diabetes between the ages of 0 to 4 years and within the years 1993–1999 in Germany, and participated in the questionnaire survey between the ages of 11 and 17 years, together with their parents or other caregivers. A total of 6,813 children and adolescents fulfilled the inclusion criteria for the comparison group: participation in the KiGGS between the ages of 11 to 17 years, together with their parents or other caregivers.

### Variables

A brief questionnaire adopted worldwide for assessing emotional and behavioral problems with child psychiatric relevance, the Strengths and Difficulties Questionnaire (SDQ), was used. The self- and parent-report versions of the SDQ consist of 25 3-point Likert-scaled items referring to the past six months. In addition, the SDQ has a 4-point Likert-scaled impact supplement to report on overall distress and social impairment of the child. The items are grouped in subscales, each containing five items: emotional symptoms, conduct problems, hyperactivity-inattention, peer problems, prosocial behavior, and impact. Each subscale score ranges from 0 to 10. Higher scores indicate greater difficulties/impact, except for prosocial behavior, where a higher score reflects more strength. A total difficulties score is calculated by totaling the four subscale scores indicating difficulties (emotional symptoms, conduct problems, hyperactivity-inattention, peer problems) [23], [24]. Norms from the United Kingdom were applied for the classification into normal, borderline, and abnormal scores [25]. Only participants with abnormal SDQ total difficulties scores were considered to have mental health problems. The German SDQ meets the basic psychometric requirements of a reliable and valid measurement; it has a good internal consistency and validity, and the five-factor model provides good fit. The items and subscales correspond to the major categories and criteria of the current psychiatric classification systems, ICD-10 and DSM-IV [26]–[29].

QoL was assessed by means of the self-report version of the Revised Children's Quality of Life Questionnaire (KINDL-R). The KINDL-R questionnaire consists of 24 5-point Likert-scaled items that cover six dimensions: physical well-being, emotional well-being, self-esteem, family, friends, and school. The six subscales were combined to form a total score. All measured values are given on scales of 0 to 100 points with higher scores indicating better QoL. The KINDL-R was evaluated to be a methodologically suitable, psychometrically sound and flexible measure to assess the QoL in children and adolescents through self-report [30]–[32].

Several covariates were included in the analyses: age, sex, socioeconomic status (integrated information about parental education level, parents' professional status, and household income classified into low, intermediate, and high according to [33]), immigrant background (yes, no), region of residence in Germany (East, West), family structure (living together with biological parents; yes, no), informant of the proxy report (mother, father, mother and father together, others), body mass index (BMI; classified into underweight, normal weight, and overweight/adiposity according to [34]), and hospitalization during the past 12 months (yes, no).

### Statistical analyses

All analyses were performed with SAS for Windows version 9.3 (SAS Institute, Cary, North Carolina, USA). The descriptive statistics are reported as percentages or means and standard deviations (SD). All analyses, including KiGGS data, were weighted with a survey weighting factor as recommended to represent the age-, sex-, regional-, and citizenship-structure of the population in Germany [35].

Both univariable and multivariable regression analyses (SAS SURVEYREG and SURVEYLOGISTIC procedures) were applied to identify differences between the patient group and the reference group. To answer the first research question, the dependent variables were abnormal SDQ scores (yes vs. no in total difficulties, emotional symptoms, conduct problems, hyperactivity-inattention, peer problems, and prosocial behavior) based on self- and proxy reports. For each outcome, two models were applied: model 1.1 (M1.1) included a term for differences between participants of the type 1 diabetes and the KiGGS study (diabetes study versus KiGGS, referred to as study effect), age group, and sex as the independent variables, and model 1.2 (M1.2) added the variables socioeconomic status, immigration background, region of residence, family structure, proxy-informant (except for SDQ self-reports), weight status, and hospitalization during the past 12 months as independent categorical variables. To answer the second research question, analyses were performed with the continuous KINDL-R total score and the KINDL-R subscale scores as dependent variables. Again, two models were used for each outcome: model 2.1 (M2.1) and model 2.2 (M2.2) were identical to M1.1 and M1.2, but additionally included a term for mental health problems (abnormal SDQ total difficulties score yes vs. no) and the interaction term study effect * mental health problems as independent variables. Two-sided p-values ≤0.05 were considered statistically significant.

## Results

### Description of the study populations

The study populations did not differ regarding the proportion of boys (54.1% in the diabetes study versus 51.3% in the KiGGS, p = 0.188). However, they differed regarding mean age (15.3 (1.7) years in the diabetes study versus 14.6 (2.0) years in the KiGGS, p<0.001), region of residence (p = 0.003), family structure (p = 0.010), socioeconomic status, immigrant background, informants of the proxy reports, BMI, and hospitalization during the past 12 months (each p<0.001) (Table 1). A more detailed description has been published elsewhere [20].

**Table 1:** Description of the two study populations.

Category	Subcategory	Diabetes study (n = 629)*	KiGGS (n = 6,813)#
Sex	Boys	54.1	51.3
	Girls	46.0	48.7
Age	11–13 years	24.0	39.6
	14–17 years	76.0	60.4
Socioeconomic status	Low	17.9	27.4
	Intermediate	48.2	47.2
	High	33.9	25.3
Immigrant background	No	98.3	82.5
	Yes	1.8	17.5
Region of residence	West	86.2	81.4
	East	13.8	18.6
Family structure	Biological parents	79.2	74.6
	Other (e.g. mother and partner, single parent, relatives)	20.8	25.4
Informants of the proxy reports	Mothers	71.7	81.0
	Fathers	6.4	11.1
	Mothers and fathers	20.8	4.7
	Others	1.1	3.2
Body Mass Index	Underweight	3.3	7.5
	Normal weight	80.7	74.8
	Overweight (incl. adiposity)	16.0	17.7
Hospitalization during last 12 months	No	72.4	92.4
	Yes	27.6	7.6

* Percentages.

# Weighted percentages.

In addition, the participants in the diabetes study were characterized by a mean type 1 diabetes manifestation age of 2.7 years (SD = 1.1, range 0.6–4.9 years), by a mean diabetes duration of 12.5 years (SD = 1.6, range 10.0–16.5 years), and by a mean hemoglobin A1c (HbA1c) of 8.3% (SD = 1.3, range 5.6–14.4%, 28.2% with HbA1c <7.5%, 25.2% with HbA1c >9.0%). A total of 48.8% of the patients used continuous subcutaneous insulin infusion, 43.3% had ≥4 daily injections, and 7.9% had 1–3 daily injections.

### Frequency of mental health problems

The proportions of adolescents with mental health problems were overall comparable in both study populations (Table 2). Based on the self-reports, the percentage of adolescents with abnormal total difficulties scores was higher in the patient group than in the KiGGS (4.4% versus 2.9%, p = 0.036). The OR for abnormal self-reported total difficulties in the patient group was 1.62 (95%-CI: 1.07–2.48) in the minimal adjusted model M1.1 and 1.61 (95%-CI: 1.01–2.56) in the fully adjusted model M1.2. However, the percentage of adolescents with abnormal scores in the four subscales indicating difficulties were not different. In addition, the percentage of adolescents with abnormal prosocial behavior was higher in the diabetes study than in the KiGGS (4.9% versus 2.9%, p = 0.007). The OR for self-reported abnormal prosocial behavior in the patient group was 1.62 (95%-CI: 1.09–2.42) in the M1.1 and 1.82 (95%-CI: 1.18–2.80) in the M1.2.

**Table 2:** Proportion of adolescents with abnormal SDQ scores in patients with type 1 diabetes compared to KiGGS participants.

SDQ Outcome	% abnormal* based on self-reports							% abnormal* based on proxy-reports						
	Diabetes study	KiGGS	p†	OR [95%CI]‡	p‡	OR [95%CI]§	p§	Diabetes study	KiGGS	p†	OR [95%CI]‡	p‡	OR [95%CI]§	p§
Total difficulties	4.4	2.9	0.036	1.62 [1.07;2.48]	0.024	1.61 [1.01;2.56]	0.044	7.9	7.2	0.718	1.14 [0.84;1.56]	0.403	1.05 [0.73;1.52]	0.788
Emotional symptoms	4.7	4.0	0.401	1.12 [0.74;1.69]	0.593	1.10 [0.71;1.72]	0.660	10.6	9.7	0.651	1.17 [0.89;1.54]	0.252	1.13 [0.83;1.54]	0.427
Conduct problems	4.4	5.3	0.319	0.78 [0.52;1.17]	0.225	0.87 [0.57;1.34]	0.531	14.3	14.2	0.728	1.01 [0.79;1.28]	0.944	1.03 [0.79;1.34]	0.848
Hyperactivity-inattention	7.0	8.3	0.239	0.85 [0.61;1.18]	0.328	0.82 [0.58;1.16]	0.253	3.5	6.5	0.011	0.57 [0.37;0.89]	0.014	0.57 [0.39;0.92]	0.023
Peer problems	3.9	3.0	0.198	1.35 [0.87;2.10]	0.182	1.33 [0.81;2.19]	0.260	11.9	12.9	0.743	0.91 [0.70;1.18]	0.467	1.01 [0.76;1.35]	0.939
Prosocial behavior	4.9	2.9	0.007	1.62 [1.09;2.42]	0.017	1.82 [1.18;2.80]	0.007	5.5	4.1	0.140	1.28 [0.88;1.87]	0.190	1.36 [0.91;2.03]	0.129
Impact	10.5	-	-	-	-	-	-	18.0	-	-	-	-	-	-

* Based on self-reports/proxy reports, abnormal was defined as a score for total difficulties ≥20/≥17, for emotional symptoms ≥7/≥5, for conduct problems ≥5/≥4, for hyperactivity-inattention ≥7/≥7, for peer problems ≥6/≥4, for prosocial behavior <5/<5, and for impact ≥2/≥2 [25].

† Chi-squared test.

‡ Diabetes study versus the reference group KiGGS adjusted for age group and sex (Model 1.1).

§ Diabetes study versus the reference group KiGGS adjusted for age group, sex, socioeconomic status, immigration background, region of residence, family structure, proxy-informant (except self-reports), weight status, and hospitalization during past 12 months (Model 1.2).

Based on the proxy reports, the percentage of adolescents with abnormal SDQ scores differed only in the hyperactivity-inattention subscale between the diabetes study and the KiGGS (3.5% versus 6.5% with abnormal score, p = 0.011). The OR for proxy-reported abnormal hyperactivity-inattention in the patient group was 0.57 (95%-CI: 0.37–0.89) in the M1.1 and 0.57 (95%-CI: 0.39–0.92) in the M1.2.

A total of 10.5% of the patients and 18.0% of their parents reported abnormal impact scores. Comparative data from the KiGGS were not available.

### Associations between mental health problems and QoL

**Figure 1:**
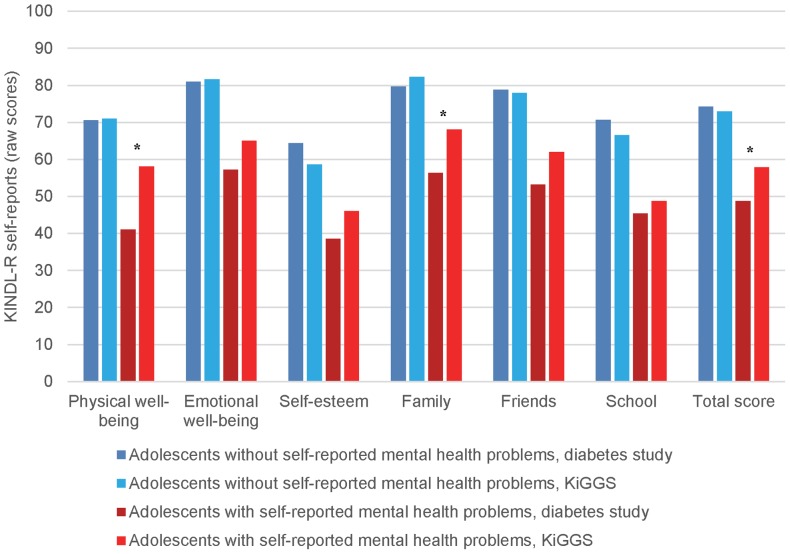
QoL in patients with type 1 diabetes compared to KiGGS participants differentiated by self-reported mental health problems. * Clinically important differences between the patient group and the reference group.

The patient group without self-reported mental health problems (defined as normal/borderline SDQ total difficulties score) rated their QoL partly better and partly worse than peers' scores (Figure 1 and Table 3). According to the M2.2, diabetic patients scored on average 4.9 points higher for the KINDL-R dimension “self-esteem”, 3.9 points higher for “school”, 1.6 points higher for “friends”, and 1.4 points higher for total QoL compared to their peers. In addition, they scored 1.9 points lower for the dimension “family”.

**Table 3:** Self-reported QoL in patients with type 1 diabetes compared to KiGGS by self-reported mental health problems.

KINDL-R	Self-reported QoL-Scores									
	Without self-reported mental health problems*					With self-reported mental health problems*				
	Diabetes study mean (SD)	KiGGS mean (SD)	p†	Study effect [95%CI] p‡	Study effect [95%CI] p§	Diabetes study mean (SD)	KiGGS mean (SD)	p†	Study effect [95%CI] p‡	Study effect [95%CI] p§
Physical well-being	70.6	71.0	0.644	0.30	0.43	41.0	58.1	<0.001	−16.63	−16.74
	(18.2)	(17.3)		[−1.20;1.80]	[−1.14;2.01]	(16.8)	(19.5)		[−23.50;−9.80]	[−24.17;−9.31]
				0.694	0.590				<0.001	<0.001
Emotional well-being	81.0	81.6	0.358	−0.13	−0.50	57.2	65.0	0.046	−7.55	−6.34
	(13.3)	(13.4)		[−1.26;1.00]	[−1.70;0.70]	(18.2)	(19.3)		[−14.82;−0.28]	[−13.98;1.31]
				0.822	0.414				0.042	0.104
Self-esteem	64.4	58.6	<0.001	5.14	4.88	38.5	46.0	0.099	−7.92	−7.09
	(18.1)	(19.4)		[3.60;6.67]	[3.29;6.47]	(16.8)	(22.3)		[−15.09;−0.76]	[−15.19;1.01]
				<0.001	<0.001				0.030	0.086
Family	79.7	82.3	<0.001	−2.07	−1.85	56.3	68.1	0.007	−11.42	−11.09
	(17.3)	(16.4)		[−3.53;−0.62]	[−3.37;−0.32]	(21.9)	(21.2)		[−20.16;−2.68]	[−20.18;−1.99]
				0.005	0.018				0.012	0.017
Friends	78.8	77.9	0.182	1.56	1.63	53.2	62.0	0.031	−8.26	−5.41
	(14.6)	(15.5)		[0.30;2.82]	[0.30;2.97]	(19.3)	(20.0)		[−15.83;−0.69]	[−13.74;2.92]
				0.015	0.016				0.032	0.203
School	70.7	66.6	<0.001	5.12	3.92	45.4	48.8	0.401	−2.56	−3.30
	(17.0)	(17.9)		[3.68;6.57]	[2.44;5.40]	(18.1)	(20.2)		[−9.86;4.74]	[−11.70;5.09]
				<0.001	<0.001				0.492	0.440
Total score	74.2	73.0	0.007	1.66	1.42	48.8	57.9	<0.001	−8.78	−8.09
	(11.1)	(10.6)		[0.74;2.58]	[0.46;2.38]	(10.2)	(12.8)		[−12.86;−4.69]	[−12.56;−3.62]
				<0.001	0.003				<0.001	<0.001

* Defined as a SDQ score for self-reported total difficulties <20 and ≥20, respectively.

† Two-sided t-test.

‡ Estimated average differences between the diabetes study and the KiGGS (reference group) adjusted for age group and sex (Model 2.1).

§ Estimated average differences between the diabetes study and the KiGGS (reference group) adjusted for age group, sex, socioeconomic status, immigration background, region of residence, family structure, weight status, and hospitalization during past twelve months (Model 2.2).

Several QoL differences between the two study populations were observed for the subgroups with self-reported mental health problems (defined as abnormal classified SDQ total difficulties score) in the univariable and M2.1 analyses (Figure 1 and Table 3). However, according to the M2.2, compared to the reference group with mental health problems, diabetic patients with mental health problems reported lower scores only in the dimensions “physical well-being” (−16.7 points) and “family” (−11.1 points), and also for total QoL (−8.1 points).

**Figure 2:**
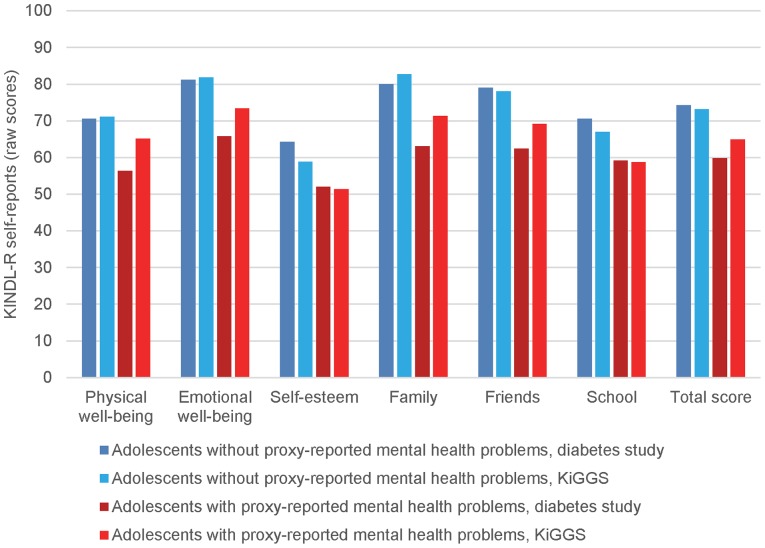
QoL in patients with type 1 diabetes compared to KiGGS participants differentiated by proxy-reported mental health problems.

The patients whose caregivers had not reported mental health problems for their children rated on average 4.0 points higher for “self-esteem”, 3.1 points higher for “school”, and 2.6 points lower for “family” than their peers from the KiGGS (Figure 2 and Table 4). The QoL of adolescents with type 1 diabetes and proxy-reported mental health problems did not differ significantly from the QoL of peers with proxy-reported mental health problems according to the M2.2, although the univariable and M2.1 analyses indicated several differences.

**Table 4:** Self-reported QoL in patients with type 1 diabetes compared to KiGGS by proxy-reported mental health problems.

KINDL-R	Self-reported QoL-Scores									
	Without proxy-reported mental health problems*					With proxy-reported mental health problems*				
	Diabetes study mean (SD)	KiGGS mean (SD)	p†	Study effect [95%CI] p‡	Study effect [95%CI] p§	Diabetes study mean (SD)	KiGGS mean (SD)	p†	Study effect [95%CI] p‡	Study effect [95%CI] p§
Physical well-being	70.6	71.1	0.471	−0.01	−0.02	56.3	65.1	0.002	−7.12	−5.45
	(18.5)	(17.3)		[−1.55;1.54]	[−1.68;1.63]	(22.0)	(18.6)		[−13.3;−0.92]	[−12.25;1.36]
				0.993	0.979				0.025	0.117
Emotional well-being	81.2	81.8	0.328	−0.22	−0.71	65.8	73.4	0.004	−6.82	−5.65
	(13.3)	(13.3)		[−1.38;0.94]	[−1.94;0.53]	(19.7)	(17.6)		[−12.30;−1.34]	[−11.41;0.11]
				0.709	0.261				0.015	0.054
Self-esteem	64.3	58.8	<0.001	4.83	4.00	52.0	51.4	0.885	0.03	0.06
	(18.4)	(19.3)		[3.25;6.42]	[2.33;5.67]	(19.7)	(21.2)		[−5.67;5.73]	[−5.85;5.96]
				<0.001	<0.001				0.991	0.985
Family	80.0	82.7	<0.001	−2.18	−2.59	63.1	71.3	0.008	−7.21	−6.96
	(17.1)	(16.1)		[−3.65;−0.70]	[−4.14;−1.04]	(22.7)	(20.3)		[−13.77;−0.65]	[−14.0;0.07]
				0.004	0.001				0.031	0.053
Friends	79.0	78.1	0.146	1.54	1.28	62.4	69.2	0.021	−5.58	−4.39
	(14.4)	(15.3)		[0.28;2.80]	[−0.08;2.65]	(20.6)	(19.7)		[−11.69;0.52]	[−10.75;2.00]
				0.017	0.065				0.073	0.177
School	70.6	67.0	<0.001	4.67	3.09	59.2	58.7	0.865	2.30	2.38
	(17.1)	(17.9)		[3.18;6.16]	[1.52;4.66]	(19.6)	(19.2)		[−3.50;8.10]	[−3.85;8.61]
				<0.001	<0.001				0.437	0.454
Total score	74.3	73.2	0.022	1.47	0.83	59.8	64.9	0.006	−4.07	−3.37
	(11.2)	(10.6)		[0.51;2.42]	[−0.17;1.83]	(14.1)	(12.0)		[−8.13;−0.02]	[−7.58;0.85]
				0.003	0.104				0.049	0.117

* Defined as a SDQ score for proxy-reported total difficulties <17 and ≥17, respectively.

† Two-sided t-test.

‡ Estimated average differences between the diabetes study and the KiGGS (reference group) adjusted for age group and sex (Model 2.1).

§ Estimated average differences between the diabetes study and the KiGGS (reference group) adjusted for age group, sex, socioeconomic status, immigration background, region of residence, family structure, weight status, and hospitalization during past twelve months (Model 2.2).

## Discussion

In this study, the frequency of mental health problems and their associations with QoL were analyzed in adolescents with early-onset and long-duration type 1 diabetes and compared with peers from the general population. The observed differences between the two study populations regarding the frequency of mental health problems were dependent on the respondent, but were generally minor (first research question): few adolescents with early-onset and long-lasting type 1 diabetes reported abnormal total difficulties and abnormal prosocial behavior (each less than 5%). The proportions were slightly higher than the prevalence of mental health problems in the general population. The proxy reports pointed to a lower proportion of adolescents with abnormal hyperactivity-inattention in the diabetes study than in the general population.

### Comparison with previous literature

Even though the findings are plausible in the context of previous research [5]–[10], one has to keep in mind the large methodological differences between studies. Results are probably highly dependent on sample characteristics, applied screening instruments, and statistical methods. All previous studies that compared adolescents with and without type 1 diabetes on indices of mental health problems had in common that the sample size was quite small, with less than 150 participants in the patient group. This might explain the fact that no [5], [6], [8], [9] or only two [7] to three [10] confounding factors were considered in these studies. However, it is known that there exists a strong association between demographic factors (e.g., age, sex, socioeconomic factors, migration) and mental health [27], [36]–[39].

### Implications

The observed associations between mental health problems and QoL (second research question) were partially distinct, but depended on the respondent and the statistical model used: based on self-reports, the QoL of adolescents with type 1 diabetes and mental health problems was more severely impaired than was the case in the general population with mental health problems (total score, dimensions “physical well-being” and “family” in the fully adjusted model). However, according to the proxy reports, such differences could not be observed after adjustment for confounding factors. Differences between self- and parent-rated SDQ have been observed before in a representative sample [40] and a clinical setting [29]. The authors concluded that the informant source of the mental health problem modifies the estimations, because a subject can be looked at from different perspectives. Usually, the use of mental health services and treatment decisions depends on parent-reports. However, the differences in perceptions should be taken into account so that the young patients receive the help they really need [40].

As far as we know, there have been no previous reports of more severe QoL impairments among adolescents with type 1 diabetes and mental health problems than among peers from the general population with mental health problems. This raises the question of whether the observed differences are of clinical importance. In absence of more specific information for the KINDL-R, we base our decision on clinical relevance of observed differences on the 0.5 SD default value as proposed by Norman et al for QoL measures used in patients with chronic diseases [41]. The differences between the diabetes study and the normative sample were higher than 0.5 SD of the normative sample for total QoL (|−8.1| >6.4) and QoL in the dimensions “physical well-being” (|−16.7| >9.7) and “family” (|−11.1| >10.6) among adolescents with self-reported mental health problems (Table 3). Thus, we conclude that only these differences are of clinical importance. The observed differences between the study populations without mental health problems are probably not clinically relevant, because the average differences were below the 0.5 SD default value.

### Limitations

This study has some limitations. A general limitation of our analysis is that the cross-sectional design prevents any inferences about causality. In addition, the SDQ is a screening tool for mental health problems, but it is not suitable for diagnosing psychiatric disorders. Although the SDQ is a widely used tool, there is still ongoing controversy on methodological issues. It has been suggested that using the broader internalizing dimension (covering emotional and peer items) and externalizing dimension (including behavioral and hyperactivity items) may be more appropriate for low-risk epidemiological samples. In addition, population-specific SDQ norms have been recommended [42]. We retained the original subscales because the five-dimensional model was supported in Germany. Additionally, we used the UK norms because German norms have not yet been established for SDQ self-reports [27], [28]. A further limitation is that the generalizability of our findings might be limited due to selection bias. An indication of this is that the patient group differed significantly from the representative sample with respect to some demographic factors. We therefore considered all of these factors as potential confounders when comparing the patient group with the representative sample. Nonetheless, there might be relevant methodological differences between the two studies for which we could not adjust. One might expect that patients with better glycemic control were more prone to participation. However, the mean reported HbA1c of our sample (HbA1c 8.3%) was no better than the mean HbA1c documented in a large database of patients with type 1 diabetes in Germany in the year 2009 (N = 30,708, age <20 years, diabetes duration >2 years, HbA1c 8.1%) [3].

Despite these limitations, there are a number of strengths of this study. In order to focus on a patient group of special concern, the analyses were based on a population-based cohort with a type 1 diabetes onset during the first five years of life. The patients were intensely treated, and therefore reflect current diabetes care in Germany [3]. Reference data came from the KiGGS which is representative of the general population in Germany. Since comprehensive individual data for patients and KiGGS participants were available, extensive statistical analyses were performed and adjusted for demographic and health-related factors. Mental health, QoL, and confounding factors were assessed with the same questions in the diabetes study as in the KiGGS to achieve maximum comparability. Mental health was assessed by a standardized, validated screening tool with two different informant sources whereby the importance of the self- and proxy reports were considered equally. This enabled us to shed light on the differences in perceptions, which can be important for treatment decisions. Only youths with abnormally high symptom scores were regarded as having mental health problems in order to provide a conservative estimate.

## Conclusions

The results of this study offer insights into the mental health of 11- to 17-year-olds with early-onset and long-duration type 1 diabetes which are relevant for clinical practice. Only a minority of patients was screened positive for mental health problems. However, these patients rated their own QoL significantly lower than one would expect from observations in the general population. All those who are engaged in diabetes care should be aware of this association between mental health and QoL. Patients with a high risk of mental health problems, and their parents, might profit from intensified psychosocial support as part of pediatric diabetes care at an early stage in order to prevent the emergence of more serious problems. Thus, early prevention and intervention as part of long-term pediatric diabetes care is recommended for patients at risk of severely impaired QoL.
